# FBXW7‐Related Neurodevelopmental Disorder: Clinical Spectrum, Molecular Mechanisms, and Tumor Predisposition

**DOI:** 10.1155/humu/3764750

**Published:** 2026-05-08

**Authors:** S. Savasta, F. F. Comisi, E. Fiumicelli, G. B. Dell’Isola, G. Di Pasquale, G. D. Mangano, L. Zagaroli, V. Salpietro, A. Verrotti

**Affiliations:** ^1^ Pediatric Clinic and Rare Diseases, Microcitemico Hospital “A. Cao”, University of Cagliari, Cagliari, Italy, unica.it; ^2^ Department of Medical Sciences and Public Health, University of Cagliari, Cagliari, Italy, unica.it; ^3^ Department of Pediatrics, University of Perugia, Perugia, Italy, unipg.it; ^4^ Saint Camillus International University of Health Sciences, Rome, Italy; ^5^ Department of Developmental Disabilities, IRCCS San Raffaele Roma, Rome, Italy; ^6^ Department of Pediatrics, University of L’Aquila, L’Aquila, Italy, univaq.it; ^7^ Department of Medicine and Surgery, University of Enna Kore, Enna, Italy, unikore.it; ^8^ European Brain Research Institute (EBRI) “Rita Levi-Montalcini”, Rome, Italy, ebri.it

## Abstract

F‐box and WD repeat domain‐containing 7 (*FBXW7*) encodes the substrate‐recognition subunit of the SCF (SKP1‐CUL1‐F‐box) E3 ubiquitin ligase complex, where it regulates proteasome‐mediated degradation of key cell cycle and developmental proteins. The aim of this review is to provide a comprehensive overview of the currently available evidence on the clinical and molecular features of *FBXW7*‐related neurodevelopmental disorder (NDD). While somatic mutations in *FBXW7* are well‐established drivers of human tumors, germline variants have only recently been linked to a distinctive neurological disorder. Reported germline variants include missense, frameshift, splice‐site, and larger structural variants, with the majority clustering in the WD40 domain and disrupting substrate recognition. Functional studies confirm impaired degradation of critical regulators such as cyclin E, MYC, and NOTCH1. Clinically, affected individuals present with early developmental delay, hypotonia, and impaired language acquisition, frequently accompanied by structural brain anomalies, craniofacial dysmorphisms, and variable growth abnormalities. Additional manifestations include congenital anomalies and broad gastrointestinal involvement. Wilms tumor (WT) has been reported in a few individuals carrying germline or constitutional mosaic *FBXW7* variants, with evidence of a second somatic hit documented in tumor tissue, supporting a rare but biologically plausible role of this gene in determining WT predisposition.

## 1. Introduction

*FBXW7* is a member of the F‐box protein family and functions as the substrate‐recognition subunit of the SCF E3 ubiquitin ligase complex, which mediates proteasome‐dependent protein degradation by ubiquitinating target proteins prior to their breakdown [1]. F‐box proteins are interchangeable components of the complex and confer substrate specificity through direct binding. *FBXW7* has been extensively studied as a tumor suppressor gene [2, 3]. Deletion of the 4q31.3 region, where the gene is located, has been detected across multiple human cancers, and point mutations within *FBXW7* also contribute to carcinogenesis. FBXW7 protein expression is reduced in melanoma, pancreatic, prostate, renal, skin, testis, thyroid, carcinoid, glioma, liver, lung, and urothelial cancers [4, 5]. More recently, the *FBXW7* gene has been shown to play an important role in several biological processes, particularly in central nervous system (CNS) development and function. In fact, the implication of this gene in neurodevelopmental disorders (NDDs) has been demonstrated through animal model studies as well as clinical and genetic findings in humans [6, 7]. A recent study, using global data‐sharing platforms, reported “de novo” and inherited germline variants in 35 patients with a neurodevelopmental syndrome. Structural modeling highlighted the involvement of conserved active residues within the FBXW7 protein [8]. Collectively, these findings underscore the need for an updated narrative review of *FBXW7*‐related brain developmental disorders to highlight the critical key features of the clinical syndrome associated with genetic defects of FBXW7.

## 2. Methods

This narrative review examined clinical, molecular, imaging, and tumor‐related aspects associated with pathogenic variants in *FBXW7*. Literature was searched in PubMed/MEDLINE and Scopus, focusing on publications in English up to September 1, 2025. Additional studies were identified through citation tracking. Search terms included “FBXW7,” “WD40,” “neurodevelopmental disorder,” “developmental delay,” “hypotonia,” “language impairment,” “Wilms tumor,” and “4q31.3.” Included sources comprised case reports, case series, and cohorts describing germline or regulatory *FBXW7* variants with clinical features, as well as functional studies providing mechanistic insights. Data were extracted on genetic findings, neurodevelopmental features, neuroradiology, somatic manifestations, and oncologic outcomes. Given the rarity of the condition and heterogeneity of the available evidence, findings were synthesized qualitatively in line with SANRA recommendations for narrative reviews [9].

## 3. Biological Role of *FBXW7*

### 3.1. Structure and Function

**Figure 1:**
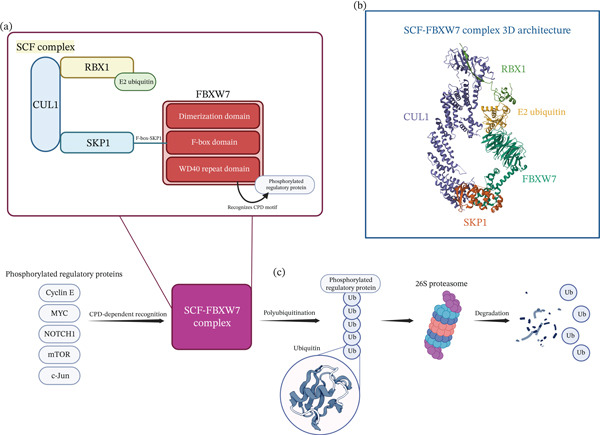
Structural architecture and proteolytic mechanism of the SCF‐FBXW7 complex in substrate recognition and degradation: (a) Schematic representation of the modular SKP1‐CUL1‐F‐box (SCF)‐F‐box and WD repeat domain‐containing 7 (*FBXW7*) complex showing Cullin 1 (CUL1) scaffold protein (light blue), RING‐box protein 1 (RBX1) RING domain (beige), E2 ubiquitin‐conjugating enzyme (light green), S‐phase kinase‐associated protein 1 (SKP1) adaptor protein (light blue), and FBXW7 F‐box protein (red) containing three functional domains: N‐terminal dimerization domain, central F‐box domain (mediating SKP1 binding), and C‐terminal WD40 repeat domain (responsible for Cdc4 phosphodegron [CPD] motif recognition in phosphorylated substrates). (b) Three‐dimensional structural representation of the assembled SCF‐FBXW7 complex (based on Protein Data Bank [PDB] 6TTU with structural editing for clarity), illustrating the spatial arrangement of complex components. Color scheme: CUL1 (purple), RBX1 (green), E2 ubiquitin (yellow), SKP1 (orange), and FBXW7 (teal). (c) Mechanistic workflow of FBXW7‐mediated substrate degradation. Phosphorylated regulatory proteins containing consensus CPD sequences, including cyclin E, MYC proto‐oncogene protein, Notch homolog 1 translocation‐associated (NOTCH1), mammalian target of rapamycin (mTOR), and transcription factor AP‐1 (c‐Jun), are specifically recognized by the FBXW7 WD40 domain through CPD‐dependent binding. Upon substrate capture, the SCF‐FBXW7 complex catalyzes formation of polyubiquitin chains on substrate lysine residues. Polyubiquitinated substrates are subsequently recognized and processed by the 26S proteasome, resulting in proteolytic degradation into short peptide fragments. This integrated pathway illustrates the critical role of SCF‐FBXW7 in maintaining cellular homeostasis through regulated turnover of key oncoproteins and cell cycle regulators. Created in BioRender. Comisi, F. (2025) https://BioRender.com/exw7oue.

FBXW7 is a protein of the F‐box family (FBX), specifically within the FBXW subgroup because it contains a tryptophan‐aspartic acid repeat domain (Figure 1). F‐box proteins are subunits of the SCF E3 ubiquitin ligase complex, a key component of the ubiquitin‐proteasome system (UPS) and a critical regulator of cell cycle progression [10]. Protein degradation through this system occurs in two sequential steps: ubiquitin chains are attached to the substrate by the coordinated action of E1 (ubiquitin‐activating enzyme), E2 (ubiquitin‐conjugating enzyme), and E3 ubiquitin ligase; the polyubiquitinated substrate is then processed and degraded by the 26S proteasome [11]. Depending on their structure, F‐box proteins determine substrate specificity and thereby regulate selective degradation. The *FBXW7* gene is located on Chromosome 4 (4q31.3). Its transcription is tightly regulated and gives rise to three isoforms: FBXW7*α*, FBXW7*β*, and FBXW7*γ*. These isoforms share 10 exons, while *β* and *γ* each include one isoform‐specific exon. FBXW7*α* is the most widely expressed, present in nearly all human tissues and localized to the nucleoplasm; FBXW7*β* is mainly expressed in the endoplasmic reticulum of the brain and in testes; and FBXW7*γ* is found in the nucleolus of the heart and muscle. These tissue‐specific expression patterns may underlie isoform‐dependent interactions and functional differences [12]. The FBXW7 protein is organized into distinct structural domains: a dimerization domain for dimer formation and substrate binding, seven tandem WD40 repeats for substrate recognition, and the F‐box domain, which mediates binding to the SKP1‐CUL1 complex and catalytic activity [13]. FBXW7 recognizes phosphorylated amino acid sequences on substrates, leading to ubiquitination. In many cases, this recognition begins with phosphorylation of the Cdc4 phosphodegron (CPD) motif by glycogen synthase kinase 3 (GSK3), followed by binding of the WD40 domain to the phosphorylated CPD. Substrates such as Notch, c‐Myc, cyclin E, Aurora, and c‐Jun are central regulators of cell cycle progression and act as oncoproteins; their accumulation, due to absent or impaired FBXW7 function, drives uncontrolled proliferation as observed in many cancers [14, 15]. Beyond its role as a tumor suppressor, FBXW7 is essential for embryonic development. *Fbxw7* knockout mice die in utero due to combined defects in hematopoietic and vascular development and impaired cardiac chamber formation [16]. FBXW7 also plays a critical role in brain development. By downregulating Notch signaling, it supports survival, positioning, and differentiation of both neurons and neural stem cells; in parallel, by interacting with c‐Jun and suppressing c‐Jun‐dependent JNK apoptotic signaling, it promotes neuronal viability [12]. These functions are further supported by evidence from *Drosophila* neuronal knockdown models, in silico protein modeling, and cell‐based functional studies [8].

### 3.2. Role in Oncogenesis

In cancer tissues, FBXW7 protein expression is reduced compared with healthy controls, as shown by immunostaining across multiple tumor types, including melanoma, pancreatic, prostate, renal, skin, testis, thyroid, carcinoid, glioma, liver, lung, and urothelial cancers [4]. Its expression and activity are regulated through genetic mutations, RNA silencing, posttranscriptional mechanisms, and homodimerization [14]. Deletion of the 4q31.3 region, the chromosomal location of the *FBXW7* gene, has been observed in more than 30% of all human cancers [17]. Point mutations also play a central role in carcinogenesis by disrupting the tumor suppressor function of *FBXW7* [18]. Somatic mutations have been extensively studied due to their impact on cancer development. They account for approximately 2.5% of mutations across all human cancers and consist mainly of single‐nucleotide variants, including missense substitutions (72.7%), nonsense substitutions (13.8%), and insertions/deletions (7.9%) [19]. The most common changes involve codons in the WD40 domain, which mediates substrate binding. In particular, R505, R465, and R479 account for about 48% of all *FBXW7* mutations [18]. These alterations disrupt interactions between FBXW7 and substrates such as cyclin E, Mcl‐1, Notch, and c‐Myc, preventing their degradation and promoting cell proliferation through persistent activation of oncogenic pathways [19]. Mutations within the CPD motifs of substrates can similarly impair recognition and binding by FBXW7 [18]. Less frequently, variants affect the F‐box domain, which mediates binding to the SKP1‐CUL1 complex, or the dimerization domain. These mutations appear to contribute less to tumorigenesis, as they are rarely detected in cancer genomes. From a functional standpoint, dimerization is not required for substrate binding, and interaction of the F‐box domain with SKP1 is robust [13]. Additional, more complex mechanisms remain under investigation, including co‐occurring mutations in *FBXW7* and genes that regulate its expression, localization, or activity [18]. Most known pathogenic variants in *FBXW7* are monoallelic. Loss of tumor suppressor activity in heterozygous cells supports haploinsufficiency as the prevailing mechanism, potentially reinforced by autoregulatory feedback through substrates such as HES5 [20]. The central role of FBXW7 in degrading proteins that maintain cellular homeostasis explains its frequent inactivation in human cancers and its importance as a tumor suppressor.

### 3.3. Role of *FBXW7* in Cellular Development and Evidence From Animal Models

*FBXW7* was identified as a potential gene involved in neurodevelopment in a recent bioinformatic analysis of a multicenter cohort [6]. Germline *FBXW7* variants have since been reported in individuals with NDDs, consistent with experimental data highlighting its role in neural cell proliferation, differentiation, and survival [8]. These effects are mediated through diverse mechanisms and substrate interactions. For example, zebrafish larvae carrying mutations in the substrate‐binding region of FBXW7 exhibited increased mTOR signaling in oligodendrocyte lineage cells and consequent hypermyelination [21]. FBXW7 also plays a central role in the lineage decision between oligodendrocytes and Schwann cells (SCs). In SC‐specific knockout mice, FBXW7 deficiency enabled SCs to myelinate multiple axons in vivo and in vitro, to generate myelin around large‐caliber axons, and simultaneously to ensheath additional small‐caliber axons without myelinating them. Increased SC numbers were observed at multiple developmental stages [22]. These phenotypes resemble those seen during peripheral nerve repair, when SCs adopt a branched morphology, likely through c‐Jun and mTOR signaling pathways [23, 24]. Beyond myelination, FBXW7 regulates neural stem and progenitor cell differentiation and viability. Hoeck et al. [12] demonstrated in *Fbxw7* knockout mice that neural stem cell differentiation was impaired and progenitor cell death increased, accompanied by accumulation of N‐terminally phosphorylated c‐Jun and active NOTCH1, two SCF‐FBXW7 substrates implicated in progenitor cell survival and stem cell differentiation [25]. Increased Notch activity secondary to FBXW7 inactivation also promoted neural stem cell death and astrocytic differentiation at the expense of neurons; treatment with Notch pathway inhibitors abrogated this effect [26]. In neural and intestinal stem cells, FBXW7‐Notch signaling participates in lateral inhibition, mediated by negative feedback from the Notch effector HES5. This reduces Notch degradation by FBXW7, creating unequal Notch levels in adjacent cells and leading to divergent differentiation outcomes [20]. These mechanisms help explain the perinatal lethality of *Fbxw7* knockout mice, characterized by abnormal brain morphology and absent suckling, which limit investigations in the adult brain but underscore the gene’s essential role in brain development [26]. Even selective inactivation of *Fbxw7* in the cerebellar anlage severely disrupted cytoarchitecture and migration, with reduced Purkinje cell numbers, defective axonal arborization, and aberrant progenitor migration, resulting in a smaller cerebellum with excess fissures. Concomitant loss of c‐Jun mitigated these effects, rescuing Purkinje cell numbers and arborization [27]. *Fbxw7* has also been implicated in neuronal death after ischemic injury. In this context, it is phosphorylated and destabilized by Cdk5, a cyclin‐dependent kinase activated through a calpain‐dependent cascade [28]. Taken together, these findings establish FBXW7 as a critical regulator of neurodevelopmental processes, acting through substrate‐specific interactions. Ongoing studies continue to refine its physiological functions and explore the pathological and therapeutic implications of its disruption.

## 4. Genetic Findings in FBXW7‐Related Neurodevelopmental Syndrome

### 4.1. Mutation Types and Distribution

**Figure 2:**
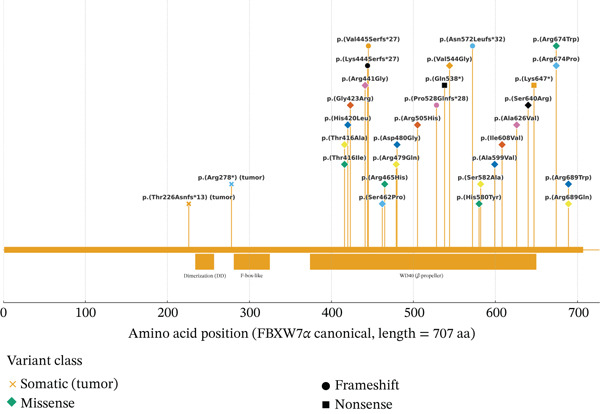
FBXW7*α* lollipop plot. FBXW7*α* (707 aa) with dimerization domain 234–257, F‐box‐like 281–325, and WD40 374–650. Stems mark variant positions; shapes indicate class: diamonds, missense; circles, frameshift; squares, nonsense; X marks, tumor‐only. Variants without amino acid coordinates (splice/CNV) are omitted; recurrent variants are shown once. Colors are decorative only and convey no meaning. A clear enrichment appears within the WD40 region.

Germline pathogenic heterozygous variants in *FBXW7* have been reported in individuals with NDDs characterized by variable phenotypes, including global developmental delay (GDD), hypotonia, moderate to severe intellectual disability (ID), and gastrointestinal manifestations. These features may occur either in combination or in isolation [8]. Reported variants include deletions, nonsense, frameshift, splice‐site, and missense changes, with both de novo and inherited variants reported in the literature (Figure 2). The most frequent are missense variants, all clustered in the carboxy‐terminal half of the protein, primarily within the WD40 domain. Three recurrent variants have been reported in unrelated individuals. Frameshift variants represent the second most common class and typically affect the longest transcript of *FBXW7*. Some of these are predicted to undergo nonsense‐mediated decay (NMD), leading to loss of function, while truncated proteins may be degraded through the UPS. Rarer alterations include large chromosomal deletions encompassing the *FBXW7* locus and splice‐site variants resulting in donor site loss [8]. No mutations have been identified in the N‐terminal region, which contains the F‐box domain, possibly reflecting lethality or subclinical phenotypes associated with such changes. Additional studies are needed to clarify FBXW7 activity in monomeric versus dimeric states in heterozygous individuals, as substrate interactions and even self‐regulation may be altered, including effects on subcellular localization (cytoplasm, nucleus, and nucleolus). Haploinsufficiency or loss of function is currently considered the most likely pathogenic mechanism [8]. More recently, copy number variations (CNVs) involving *FBXW7* at the 4q31.3 locus have been investigated for their role in NDDs. Deletions or duplications in this region may disrupt the composition of topologically associating domains (TADs) within the regulatory region of the *FBXW7* gene, leading to transcriptional dysregulation [29]. Supporting evidence comes from functional and RNA sequencing studies, which demonstrated altered *FBXW7* expression in 4q31.3 deletion carriers compared to age‐ and sex‐matched healthy individuals [29].

### 4.2. Functional Consequences

*FBXW7*‐related NDD shares mechanistic features with other UPS‐related syndromes, including CUL4B‐associated Cabezas syndrome (OMIM #300354) [30], PSMD12‐related Stankiewicz‐Isidor syndrome (OMIM #617516) [31], and FBXO11‐related intellectual developmental disorder with dysmorphic facies and behavioral abnormalities (OMIM #618089) [32], in which disrupted protein degradation contributes to overlapping manifestations such as developmental delay (DD), hypotonia, and language impairment. However, FBXW7 pathogenic variants seem to show a particular impact on cerebellar development and cluster in the WD40 domain, highlighting both shared and distinct pathogenic aspects. Computational studies have shown that reported missense variants alter amino acid residues critical for the substrate‐binding interface of FBXW7 [8]. These variants differ from missense changes identified in the general population, which are typically located away from the substrate‐binding interface of the FBXW7 protein. Variants affecting the WD40 domain have a greater impact on FBXW7 function than those occurring outside this domain, particularly by impairing the degradation of cyclin E1 and cyclin E2, resulting in elevated levels of both proteins in vivo. An exception is the Asp480Gly variant, which was associated with increased cyclin E2 and decreased cyclin E1 levels [8]. A similar mechanism may be hypothesized for other substrates. Regulatory processes involving *FBXW7* are complex and remain incompletely understood. They include feedback from FBXW7 substrates themselves, as well as alterations in the regulatory regions of *FBXW7* that can affect expression [29]. Regulation occurs at transcriptional, translational, and posttranslational levels. Transcription can be suppressed by CAAT/enhancer‐binding protein and by the bHLH transcription factor HES5, both of which interact with the promoter region. Another inhibitory mechanism involves methylation of CpG sites in the promoter. Conversely, p53 promotes *FBXW7* transcription, with a p53‐binding site identified in the first exon [7]. At the translational level, multiple microRNAs (miRNAs) interact with the 3 ^′^ untranslated region of *FBXW7* mRNA, reducing protein production. This effect can be counteracted by long noncoding RNAs (lncRNAs) acting as miRNA “sponges” [7]. Finally, posttranslational regulation involves ubiquitination, phosphorylation, and dimerization. Ubiquitination precedes proteasomal degradation, and FBXW7 autoubiquitination may be promoted by COP9 signalosome complex subunit 6 or inhibited by deubiquitinating enzymes such as ubiquitin‐specific peptidase 28. Additional kinases can modulate FBXW7 stability and activity through interactions that influence its dimerization state [8].

## 5. Clinical Spectrum

### 5.1. Neurological and Neurodevelopmental Features

*FBXW7* was first implicated in NDDs in 2020, when it was identified among 28 genes significantly enriched for de novo pathogenic variants through large‐scale bioinformatic analyses, although no phenotypic data were reported at that time [6]. In 2022, Stephenson et al. [8] provided the first detailed clinical description of *FBXW7*‐related NDD, also designated as DEDHIL (developmental delay, hypotonia, and impaired language; OMIM #620012), an autosomal dominant condition primarily affecting early cognitive and motor development. In this cohort of 35 individuals, neurodevelopmental abnormalities were present in 97.1%. Mild to moderate DD or ID was observed in 77.1%, while 8.6% had severe GDD or ID. Less common presentations included isolated speech and language delay (2.9%) and specific learning disabilities (5.7%). Only one individual (2.9%) showed a normal neurodevelopmental profile [8]. Hypotonia was reported in 62.9%, while seizures occurred in 22.9%. Ataxia and developmental regression were infrequent. Additional case reports have expanded the phenotypic spectrum to include hypertonia, severe expressive language delay, occasional reduced muscle strength, and cortical electrophysiological abnormalities. Structural variants at 4q31.3 involving the *FBXW7* regulatory region have been associated with similar neurodevelopmental features. Rarely, a Wilms tumor (WT) has been observed in the setting of a germline *FBXW7* variant with a somatic second hit [29]. While the overall clinical picture is relatively uniform among individuals with pathogenic *FBXW7* variants, there remains interindividual variability in neurological comorbidities and severity.

### 5.2. Neuroradiological Findings

Neuroimaging data from individuals with *FBXW7*‐related NDD indicate a recurrent pattern of structural brain anomalies, particularly affecting midline and posterior fossa structures. In the largest published cohort, Stephenson et al. [8] reported imaging in 17 individuals, with abnormalities in 13 (76.5%). The corpus callosum was absent, hypoplastic, or dysplastic in seven cases (41.2%). Cerebellar anomalies were seen in five individuals (29.4%), while delayed myelination, brainstem thickening, and polymicrogyria were each observed in 11.7%. One subject showed scattered subcortical calcifications on CT. In a detailed neuroimaging review of 10 MRI scans from seven individuals, cerebellar enlargement or borderline megacerebellum was the most frequent posterior fossa finding [8]. A patient carrying an additional familial *CACNA1A* pathogenic variant exhibited severe cerebellar atrophy, enlarged folia, and a thickened, dysmorphic brainstem and corpus callosum [8]. Further observations have been reported in single case studies. Wang et al. [33] described an infant with bilateral intraventricular and subarachnoid hemorrhage on cranial CT at Day 6 of life, accompanied by diffusely reduced white matter density; subsequent MRI confirmed persistent intraventricular blood, subarachnoid hemorrhage, and mild hydrocephalus. In a patient with a 4q31.3 duplication involving *FBXW7*, MRI revealed a lesion adjacent to the right lateral ventricular trigone, consistent with sequelae of antenatal or perinatal hemorrhage [29]. Collectively, the available imaging evidence supports a role for *FBXW7* in neurodevelopmental processes affecting commissural tracts, white matter integrity, and hindbrain architecture. However, systematic neuroradiological evaluation in larger cohorts is required to define the radiological signature of this disorder more precisely.

### 5.3. Somatic Manifestations

A broad clinical spectrum has been reported in individuals with *FBXW7*‐related neurodevelopmental syndrome, involving craniofacial morphology, growth parameters, visceral organs, and tumor predisposition. Although no pathognomonic sign has been identified, several recurrent features may suggest a recognizable syndromic profile.

#### 5.3.1. Dysmorphisms

Craniofacial anomalies are among the most frequently described somatic features. In the cohort reported by Stephenson et al. [8], no consistent facial gestalt was observed, although deep‐set eyes with upper eyelid fullness were present in 25.7% of cases (9/35). Cleft or high‐arched palate occurred in 28.6% (10/35), including overt and submucous forms. Other features included strabismus (14.3%, 5/35), refractive errors (17.1%), astigmatism (2.9%), and cerebral visual impairment (2.9%). Less common findings were midface retrusion with Class III malocclusion (2.9%, 1/35) and a tall or broad forehead (11.4%, 4/35). One individual with somatic mosaicism exhibited cutaneous Blaschkoid dyspigmentation [8]. Additional dysmorphic traits have been noted in single case reports. Wang et al. [33] described a male infant with a prominent forehead, ocular hypertelorism, and a low nasal bridge; this patient also displayed generalized overgrowth. Meier‐Abt et al. [34] reported epicanthus and broad distal phalanges, while Saito et al. [35] described a distinctive facial gestalt characterized by deep‐set eyes, upper eyelid fullness, long eyelashes, strabismus, full cheeks, upturned nares, and smooth philtrum. Patients with CNVs involving 4q31.3 also showed multisystem involvement [29]. One 5‐year‐old male exhibited mild frontal bossing, short philtrum, underdeveloped nasolabial folds, right thumb polydactyly, and two café‐au‐lait macules. A 2‐month‐old male had a wide interocular distance, marked antihelix stem prominence, and bilateral single palmar creases [29].

#### 5.3.2. Growth Parameters and Head Circumference

Abnormal head size is another recurrent feature of *FBXW7*‐related NDD. In the largest cohort to date, Stephenson et al. [8] reported macrocephaly in 28.6% (10/35) and microcephaly in 5.7% (2/35). Wang et al. [33] described a male infant with generalized overgrowth, including birth weight and length above +3 standard deviations (SD) and head circumference between +1 and +2 SD. In contrast, the patient reported by Meier‐Abt et al. [34] was born with weight and length at the 50th and 40th percentiles and head circumference at the 80th percentile, but by age 7.5 years, his head circumference had decreased to the 9th percentile, indicating postnatal deceleration of head growth. Growth data from two patients with CNVs at 4q31.3 also highlight variability. One individual had near‐average birth parameters (weight −0.35 SD, length +0.06 SD, and head circumference −0.36 SD), with head circumference increasing to +0.31 SD by age 5 years [29]. The second, a 2‐month‐old male, had consistently low measurements: birth weight −1.18 SD, length −0.63 SD, and head circumference −0.28 SD [29]. These findings reflect a heterogeneous auxological profile, with some individuals presenting overgrowth, others presenting borderline microcephaly, and some showing postnatal deceleration. Although no consistent growth pattern has been established, alterations in head circumference, ranging from macrocephaly to relative deceleration, appear to be part of the phenotypic spectrum.

#### 5.3.3. Visceral, Cardiac, and Gastrointestinal Anomalies

Visceral and organ‐related anomalies are variably represented in individuals with *FBXW7*‐related NDD. In the cohort described by Stephenson et al. [8], cardiac anomalies were present in 31.4% (11/35), including structural heart defects. Feeding difficulties were reported in 45.7% (16/35), with five patients requiring nasogastric tube support. Gastrointestinal symptoms included constipation (45.7%) and gastroesophageal reflux (20.0%), while recurrent pneumonia was observed in 8.6% (3/35). Additional anomalies have been documented in case reports. Wang et al. [33] described hydrocele and mild anemia in a male infant, who also experienced neonatal complications such as intracranial infection and hyperbilirubinemia. Meier‐Abt et al. [34] reported multiple congenital anomalies, including diaphragmatic hernia, intestinal malrotation, pectus excavatum, cryptorchidism, and inguinal hernia. Cryptorchidism was also observed in 19.2% (5/26 males) of the Stephenson et al. [8] cohort, highlighting its relevance as a recurrent manifestation. Detailed clinical and genetic features of the reported patients are summarized in Table 1.

**Table 1:** Clinical and genetic features in individuals with *FBXW7* pathogenic variants and neurodevelopmental disorders.

References	Sex	Age	Variant/domain	Inheritance	Facial dysmorphism	Neurodevelopment	Neurological features	Cardiac features	Genitourinary features	Gastrointestinal features	Malignancy
Stephenson et al. [8]	M	11y	c.1331_1332del; p.(Lys444Serfs∗27) WD40	De novo	Macrocephaly, broad forehead, high nasal root, deep‐set eyes, cleft palate	GDD, ID	Hypotonia, tactile issues	Bicuspid aortic valve, PDA surgery	Multicystic kidney, cryptorchidism	Feeding difficulties, chronic constipation	No
Stephenson et al. [8]	M	3y 2m	c.1332dup; p.(Val445Serfs∗27) WD40	De novo	Macrocephaly, epicanthus, thick eyebrows, synophrys, deep‐set eyes, high palate	GDD, language impairment	Hypotonia	Atrial defect	Inguinal testis	Neonatal sucking difficulties	No
Stephenson et al. [8]	F	14y 9m	c.1713_1714del; p.(Asn572Leufs∗32) WD40	Paternal	Deep‐set eyes, cleft palate, malocclusion	GDD, ID, language impairment, motor impairment	Hypotonia	No	No	Feeding difficulties	No
Stephenson et al. [8]	F	11y 9m	c.1713_1714del; p.(Asn572Leufs∗32) WD40	Paternal	Cleft palate	Language impairment, motor delay, ASD	No	No	No	Hyperphagia, constipation	No
Stephenson et al. [8]	F	6y 3m	c.1713_1714del; p.(Asn572Leufs∗32) WD40	Paternal	Macrocephaly, deep‐set eyes, periorbital fullness	Language impairment, motor delay, ASD	No	No	No	Hyperphagia	No
Stephenson et al. [8]	M	44y 6m	c.1713_1714del; p.(Asn572Leufs∗32) WD40	N.A.	Macrocephaly, midface retrusion, malocclusion	Limited speech	No	No	No	No	No
Stephenson et al. [8]	M	9y	c.1939A>T; p.(Lys647∗) WD40	De novo	No	Language delay, mild ID	No	No	No	No	No
Stephenson et al. [8]	F	5y	c.1236+2T>A WD40	De novo	Prominent forehead, hypertelorism, low‐set ears	Language impairment, ID, motor delay	No	No	No	No	No
Stephenson et al. [8]	M	7y 2m	Gene deletion	De novo	Facial asymmetry, low‐set ears, thin upper lip	Language delay, walk delay	No	No	No	No	No
Stephenson et al. [8]	M	2y 2m	Gene deletion	De novo	Flat occiput, hypertelorism, micrognathia	GDD, language impairment (few words), motor delay	Hypotonia	No	No	Sucking and swallowing difficulties	No
Stephenson et al. [8]	M	18y	c.1247C>T; p.(Thr416Ile) WD40	De novo	Mild, not specified dysmorphisms	ID, motor impairment	Epilepsy	No	No	No	No
Stephenson et al. [8]	F	3y	c.1246A>G; p.(Thr416Ala) WD40	Maternal	Microcephaly, highly arched eyebrows, synophrys	GDD, speech delay, walk delay	Ataxic gait	VSD	No	No	No
Stephenson et al. [8]	M	10y 2m	c.1259A>T; p.(His420Leu) WD40	De novo	Macrocephaly	No	Early hypotonia	No	No	Severe GERD, requiring G‐tube and Nissen fundoplication	No
Stephenson et al. [8]	M	14y	c.1267G>A; p.(Gly423Arg) WD40	De novo	Macrocephaly, highly arched eyebrows, synophrys, hypotelorism, high palate	GDD, limited speech (few words), ASD	Epilepsy, hypotonia	Interrupted aortic arch, multiple VSDs, subaortic stenosis	Cryptorchidism	GERD requiring G‐tube, constipation	No
Stephenson et al. [8]	M	15y	c.1267G>A; p.(Gly423Arg) WD40	De novo	Macrocephaly, low‐set ears, small mouth, high palate	GDD, ID	Hypotonia	No	Nephromegaly	Constipation	No
Stephenson et al. [8]	M	5y	c.1321C>G; p.(Arg441Gly) WD40	De novo	Macrocephaly, short nose, downturned corners of the mouth	GDD, limited speech	Hypotonia	No	Nocturia	Feeding difficulties, GERD, constipation	No
Stephenson et al. [8]	M	N.A.	c.1384T>C; p.(Ser462Pro) WD40	De novo	Macrocephaly, deep‐set eyes	Motor impairment	Hypotonia	No	No	Feeding difficulties, constipation	No
Stephenson et al. [8]	F	9y 6m	c.1394G>A; p.(Arg465His) WD40	De novo	Macrocephaly, long face, rotated ears, high narrow palate	GDD	Hypotonia	PDA surgery	No	No	No
Stephenson et al. [8]	M	6y	c.1436G>A; p.(Arg479Gln) WD40	De novo; postzygotic (mosaic: 14%)	Mild dysmorphic facial metopic ridge, epicanthal folds	GDD, limited speech (few words), motor impairment	Hypotonia	No	No	Constipation	No
Stephenson et al. [8]	M	16y	c.1439A>G; p.(Asp480Gly) WD40	De novo	Long palpebral fissures, dimpled lower lip	GDD, limited speech (few words), nonambulatory	Epilepsy, hypotonia	No	Cryptorchidism	GERD, constipation	No
Stephenson et al. [8]	M	3y	c.1514G>A; p.(Arg505His) WD40	De novo	Macrocephaly, epicanthal folds, deep‐set eyes, arched palate	Speech delay, motor delay	Hypotonia	No	No	GERD requiring G‐tube	No
Stephenson et al. [8]	F	1y 11m	c.1631T>G; p.(Val544Gly) WD40	De novo	Broad nose, small mouth, high palate	GDD	Progressive spasticity	Persistent left superior vena cava to coronary sinus	No	No	No
Stephenson et al. [8]	M	3y	c.1738C>T; p.(His580Tyr) WD40	De novo	Mild, not specified dysmorphisms	GDD, walk delay, language impairment	Hypotonia	No	No	Constipation	No
Stephenson et al. [8]	F	6y	c.1744T>G; p.(Ser582Ala) WD40	De novo	Deep‐set eyes, anteverted ears, depressed nasal bridge	GDD, walk delay	No	Atrial defect	No	No	No
Stephenson et al. [8]	M	1y 10m	c.1796C>T; p.(Ala599Val) WD40	De novo (mosaic: 23.3%)	Prominent nasal bridge	GDD, walk delay, speech delay	Epilepsy, hypotonia	No	No	Feeding difficulties	No
Stephenson et al. [8]	M	3y	c.1877C>T; p.(Ala626Val) WD40	De novo	Epicanthal folds, depressed nasal bridge	GDD, walk delay, speech delay	Epilepsy, hypotonia	No	No	Diarrhea	No
Stephenson et al. [8]	F	7y	c.1920C>A; p.(Ser640Arg) WD40	De novo	Synophrys, bilateral ptosis	GDD, nonverbal, nonambulatory	Epilepsy, hypotonia	VSD	No	GERD requiring G‐tube	No
Stephenson et al. [8]	M	11y	c.1822A>G; p.(Ile608Val) WD40	De novo	No	ID	Epilepsy	No	Incontinence	No	No
Stephenson et al. [8]	M	2y	c.2021G>C; p.(Arg674Pro)	De novo	Depressed nasal bridge, arched palate	Moderate DD, walk delay	Hypotonia	No	No	Feeding difficulties, GERD, constipation	No
Stephenson et al. [8]	M	15y	c.2020C>T; p.(Arg674Trp)	De novo	Deep‐set eyes, prominent lips	GDD, walk delay, speech delay	Ataxic gait	No	No	No	No
Stephenson et al. [8]	M	14y	c.2020C>T; p.(Arg674Trp)	De novo	No	GDD, nonverbal, walk delay	Epilepsy, hypotonia, ataxia	No	No	No	No
Stephenson et al. [8]	M	2y	c.2066G>A; p.(Arg689Gln)	De novo	Macrocephaly, deep‐set eyes, epicanthal folds, prominent ears	Speech delay	No	Bicuspid aortic valve	Cryptorchidism	GERD	No
Stephenson et al. [8]	M	10y	c.2065C>T; p.(Arg689Trp)	De novo	Small mouth	GDD, nonverbal, walk delay	Hypotonia	No	No	Constipation	No
Stephenson et al. [8]	M	12y	c.2065C>T; p.(Arg689Trp)	De novo	Macrocephaly, small ears	Moderate DD	Hypotonia	No	No	Constipation	No
Stephenson et al. [8]	M	7y	c.2065C>T; p.(Arg689Trp)	De novo	Macrodolichocephaly, ogival palate, chin hypoplasia	GDD, limited speech	Hypotonia	No	Dysplastic kidney	Constipation	No
Meier‐Abt et al. [34]	M	8y 6m	c.862‐2A>C + c.677del/p.(Thr226Asnfs∗13) in tumor tissue	De novo; mosaic	Epicanthus	GDD, ID, speech delay	Hypotonia	No	Cryptorchidism	Intestinal malrotation	Yes, WT
Saito et al. [35]	M	3y	c.1583del; p.(Pro528Glnfs∗28) WD40 + c.832C>T; p.(Arg278∗) in tumor tissue	Maternal	Microcephaly, deep‐set eyes, smooth philtrum	GDD, speech delay	Hypotonia	No	No	No	Yes, WT
Saito et al. [35]	F	4y	c.1583del; p.(Pro528Glnfs∗28) WD40	Maternal	N.A.	GDD	Hypotonia	No	No	No	No
Wang et al. [33]	M	2y 5m	c.1612C>T; p.(Gln538∗) WD40	De novo	Prominent forehead, hypertelorism, low nasal bridge	ID, speech delay	Hypertonia	No	No	No	No
Zhou et al. [29]	M	5y	4q31.3 deletion, with FBXW7 disruption	De novo	Frontal bossing, short philtrum	GDD, motor impairment, speech delay	N.A.	No	No	No	No
Zhou et al. [29]	M	2m	4q31.21q31.3 duplication, with FBXW7 disruption	De novo	Hypertelorism	DD	Hypertonia	No	No	No	No

*Note:* Updated from Stephenson et al. [8].

Abbreviations: ASD, autism spectrum disorder; DD, developmental delay; GDD, global developmental delay; GERD, gastroesophageal reflux disease; ID, intellectual disability; N.A., not available; PDA, patent ductus arteriosus; VSD, ventricular septal defect; WT, Wilms tumor.

## 6. FBXW7 and Wilms Tumor: Clinical and Molecular Aspects

### 6.1. Case Descriptions

WT has been described in two individuals with pathogenic *FBXW7* variants, suggesting a potential tumor predisposition in the context of this neurodevelopmental syndrome. The first case, reported by Meier‐Abt et al. [34], involved a 9‐year‐old boy who developed a left‐sided WT at age 7. Genetic testing identified a mosaic *FBXW7* splice‐site variant (c.862‐2A>C) predicted to be deleterious. Molecular analysis of tumor tissue revealed a somatic frameshift mutation (c.677del, p.(Thr226Asnfs∗13)), consistent with a second hit. Treatment included neoadjuvant chemotherapy, surgical resection, and adjuvant chemotherapy, with no evidence of recurrence. The second case, described by Saito et al. [35], concerned a 2‐year and 3‐month‐old boy diagnosed with Stage II mixed‐type nephroblastoma. This patient harbored a maternally inherited heterozygous variant in *FBXW7* (c.1583del, p.(Pro528Glnfs∗28)), and in tumor tissue, an additional (somatic) nonsense variant (c.832C>T, p.(Arg278∗)) was also identified. This somatic change was previously reported in colorectal cancer and WT [5, 36]. Notably, the patient’s sister, who carried the same germline variant, exhibited NDD but did not develop a tumor. Following surgery and chemotherapy, the affected boy remained disease‐free at 6 years of age.

### 6.2. The “Two‐Hit” Model

*FBXW7* encodes the substrate‐recognition subunit of an SCF‐type E3 ubiquitin ligase complex that mediates proteasome‐dependent degradation of several oncogenic proteins, including cyclin E, MYC, and NOTCH1, thereby exerting tumor suppressor activity [5]. In 2019, Mahamdallie et al. [37] identified *FBXW7* as a WT predisposition gene. In both reported cases of *FBXW7*‐related NDD with nephroblastoma, molecular analysis of tumor samples revealed evidence of a second‐hit event in the tumor, consistent with Knudson’s [38] two‐hit model of tumorigenesis. In the case described by Meier‐Abt et al. [34], a de novo constitutional mosaic splice‐site variant (approximately 35% mosaicism in blood) was followed by the acquisition of a somatic truncating variant detected exclusively in tumor tissue. In the case reported by Saito et al. [35], a constitutional pathogenic variant inherited from the unaffected mother (also present in the proband’s sister with DD) was combined with a somatic nonsense variant on the paternal allele, detected exclusively in tumor DNA, thereby confirming tumor‐restricted biallelic loss of function. Importantly, all reported mutations disrupted the WD40 domain, a highly conserved motif required for substrate binding and E3 ligase activity, thereby abolishing FBXW7 tumor suppressor function.

### 6.3. Clinical Implications

Although only two cases of WT associated with pathogenic *FBXW7* variants have been reported, these observations raise the possibility that FBXW7 inactivation may contribute to nephroblastoma predisposition. Based on these findings, Saito et al. [35] suggested that surveillance protocols may be warranted. While no international guidelines currently exist for *FBXW7*‐related cancer surveillance, renal ultrasound screening may be considered a precautionary approach, although the supporting evidence remains limited. The overall penetrance of WT appears low, but additional cases are required to better define absolute risk and to determine whether routine monitoring should be recommended.

## 7. Diagnostic and Clinical Management Considerations

The diagnosis of *FBXW7*‐related NDD requires a high index of suspicion, especially in individuals presenting with GDD, hypotonia, delayed language acquisition, and subtle but recurrent somatic features such as craniofacial dysmorphisms, congenital malformations, or macrocephaly. Given the phenotypic overlap with other neurodevelopmental syndromes, molecular confirmation is essential. According to the 2021 evidence‐based guideline of the American College of Medical Genetics and Genomics, whole‐exome sequencing (WES) represents the most effective first‐tier diagnostic approach, as it allows the detection of pathogenic or likely pathogenic variants in coding regions where most of the known pathogenic variants have been identified [39]. In cases with negative WES results but a compatible phenotype, CNVs affecting the 4q31.3 locus may be responsible [29]. In such instances, chromosomal microarray analysis should be performed if not already incorporated into the WES pipeline.

Once a molecular diagnosis is established, comprehensive clinical management is required. Given the high prevalence of neurological comorbidities, early multidisciplinary intervention, including physical, speech, and occupational therapy, should be prioritized in the first years of life [8]. Routine assessments of cognitive and motor development are recommended to guide individualized care plans. Because of the tumor suppressor role of FBXW7 and the reported cases of WT arising through a two‐hit mechanism, oncological surveillance may be considered [34, 35]. Abdominal ultrasound screening during early childhood may be considered in germline *FBXW7* carriers after shared decision‐making with the family, given the two reported WT cases and the limited evidence currently available. Optimal care should involve a multidisciplinary team, ideally coordinated through specialized clinical genetics services. In addition to pediatric neurologists and rehabilitation specialists, input from cardiologists, nephrologists, and gastroenterologists may be necessary depending on associated comorbidities (e.g., cardiac anomalies, cryptorchidism, and feeding difficulties). Patients with notable somatic features may also require otolaryngologic and ophthalmologic evaluations. Genetic counseling is an essential component of management. Although most variants appear to occur de novo, cases of parental mosaicism and mildly affected transmitting parents have also been reported [8]. Segregation analysis in parents is therefore recommended for recurrence risk assessment and reproductive planning. Families may benefit from discussion of reproductive options, including prenatal diagnosis or preimplantation genetic testing. Finally, long‐term follow‐up into adolescence and adulthood is advised. As new data emerge, recommendations for diagnosis, surveillance, and management will need periodic reassessment to reflect the evolving understanding of this disorder.

## 8. Limitations and Future Directions

While one cohort study has provided detailed phenotypic and molecular data for 35 individuals with *FBXW7*‐related NDD, most additional reports consist of individual case studies, often lacking comprehensive clinical, radiological, or longitudinal information. As a result, important aspects of the syndrome, including age‐related evolution of symptoms, penetrance of associated anomalies, and genotype–phenotype correlations, remain incompletely defined. Moreover, data on neuroimaging, oncological risk, and adult outcomes are limited. There is a clear need to systematically collect and report additional cases using standardized protocols that include detailed clinical descriptions, molecular findings, and neuroimaging data. Establishing an international registry and promoting collaboration across centers could be critical to better define the full phenotypic spectrum, clarify genotype–phenotype correlations, assess long‐term outcomes, and develop evidence‐based recommendations for diagnosis, surveillance, and management.

## 9. Conclusions

*FBXW7*‐related NDD is emerging as a distinct clinical entity defined by early DD, hypotonia, language impairment, and recurrent brain malformations. Pathogenic variants occur predominantly within the WD40 domain, supporting a central role for disrupted substrate recognition in disease mechanisms. The occurrence of WT in two individuals, both with evidence of biallelic inactivation, may suggest a biologically plausible though rare tumor predisposition. Overall knowledge remains limited, as most data derive from a single cohort and isolated case reports. Larger, systematically collected series are required to delineate genotype–phenotype correlations, clarify long‐term prognosis, and define evidence‐based management and surveillance strategies.

## Funding

No funding was received for this manuscript. Open access publishing is facilitated by Universita degli Studi di Cagliari, as part of the Wiley‐CRUI‐CARE agreement.

## Conflicts of Interest

The authors declare no conflicts of interest.

## Data Availability

Data sharing is not applicable to this article as no datasets were generated or analyzed during the current study.
